# Supplementary material for: Peptidomimetic blockade of MYB in acute myeloid leukemia
Source: Nat Commun. 2018 Jan 9;9:110. doi: 10.1038/s41467-017-02618-6 (PMC5760651; doi:10.1038/s41467-017-02618-6)
Supplement: Supplementary file 1 — Supplementary Information [file 41467_2017_2618_MOESM1_ESM.pdf]

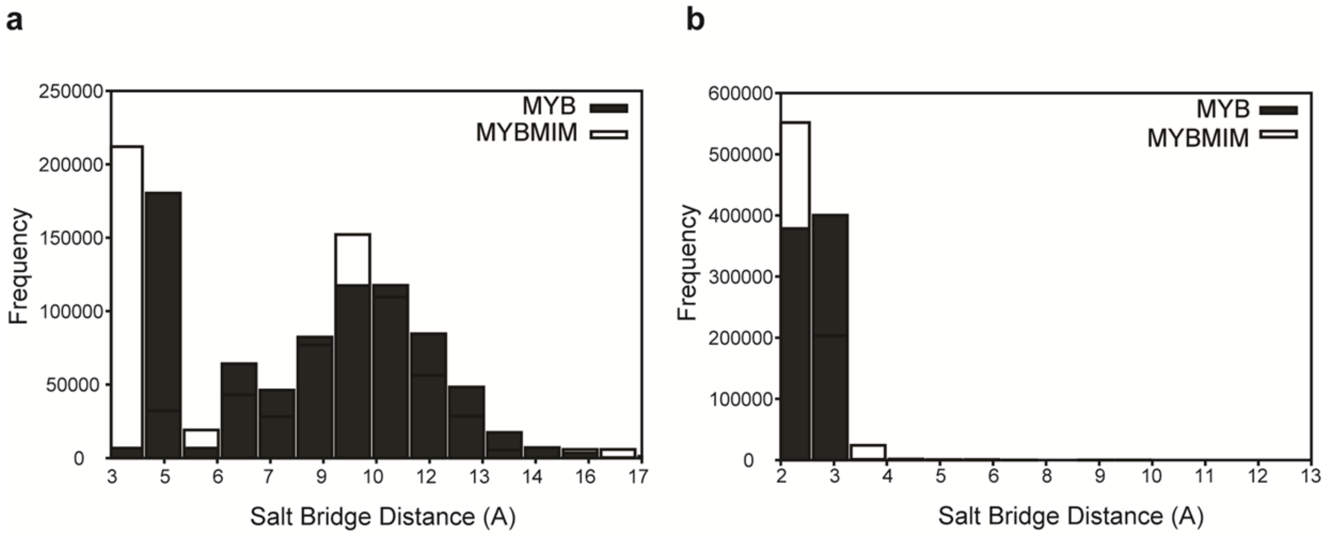

Supplementary Figure 1. Histograms of the distributions of distances of MYB:CBP for **(a)** R294:E665 and **(b)** E308:H602 salt bridges, as analyzed using replica exchange molecular dynamics simulations of the complexes of the CBP KIX domain with MYB and MYBMIM peptides.

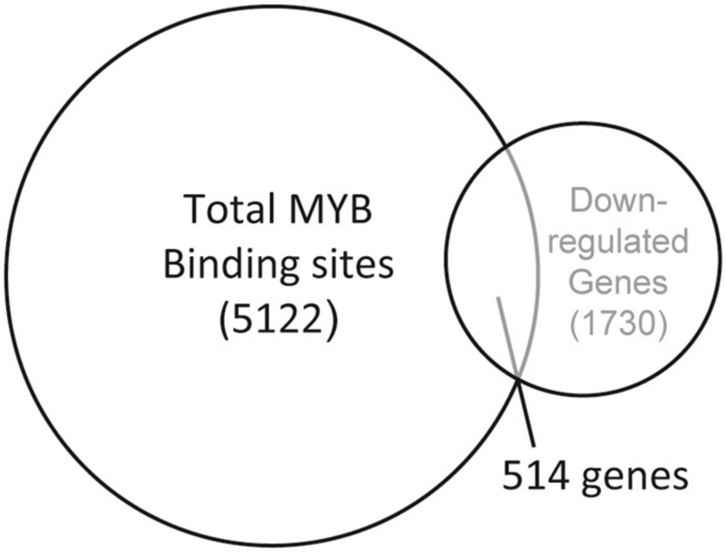

Supplementary Figure 2. Venn diagram representing the cross-section of the 5,122 MYB binding sites identified in ChIP-seq analysis as compared against the 1,730 down-regulated genes seen in RNA-seq gene expression data. 514 genes are identified in the intersection of MYB binding sites that occur within 50kb of down-regulated genes (29.7%).

Downregulated	Motif	Fold change	p-value
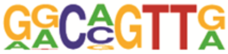	MYB	5.6	1e-145
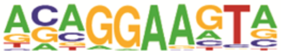	ERG	2.5	1e-38
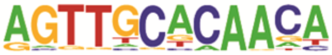	CEBPA	9.9	1e-12
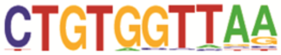	RUNX1	6.3	1e-12
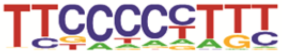	Sp1/PU.1	4.3	1e-12
Upregulated			
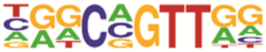	AMYB/MYBL1	4.5	1e-100
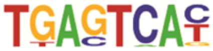	JUNB	3.3	1e-14
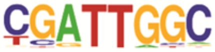	NFY	3.2	1e-11
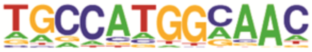	RFX1	2.7	1e-10
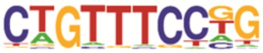	FOXO1	2.3	1e-9

Supplementary Figure 3. Enrichment of top 5 transcription factor motifs in down-regulated and up-regulated MYB peaks upon MYBMIM treatment. Motifs are sorted based on p-values and fold change (% peaks/% in background) as indicated.

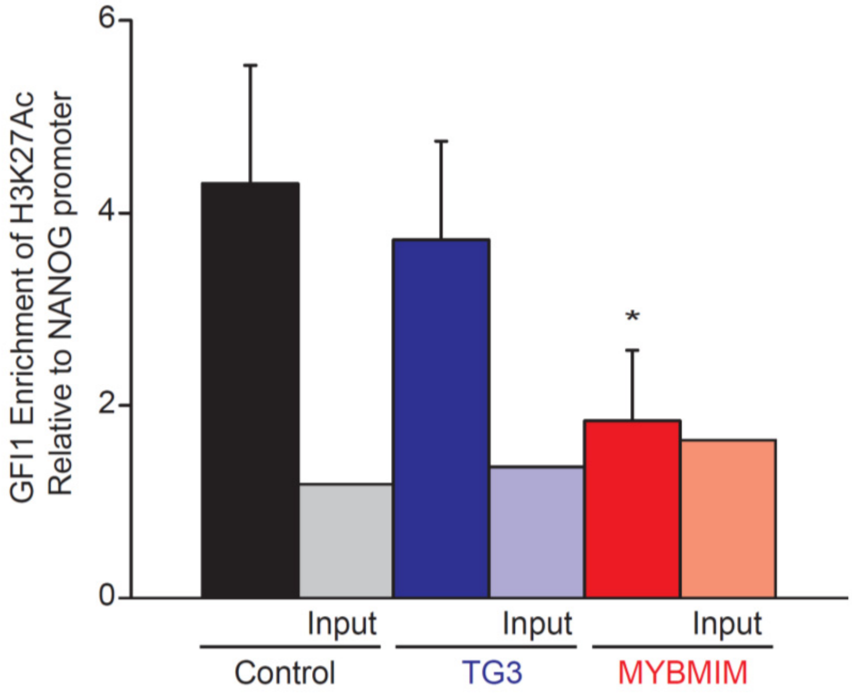

Supplementary Figure 4. Analysis of relative enrichment of H3K27Ac at the BCL2 enhancer locus compared to NANOG, as measured by ChIP-PCR upon treatment with control PBS (black), 20 μM TG3 (blue), and 20 μM MYBMIM (red) for 24 hours. Error bars represent standard deviations of three biological replicates. * $p = 0.04$ when compared to untreated control.

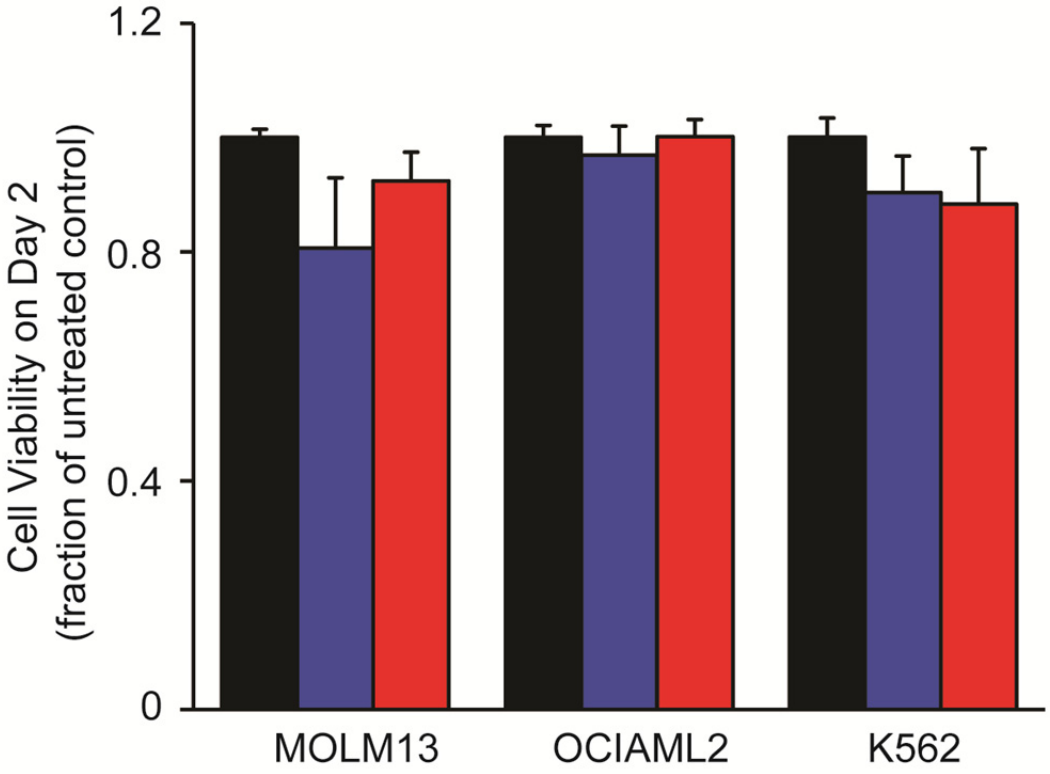

Supplementary Figure 5. Viability of MOLM13, OCIAML2, and K562 cells, treated for 2 days with control PBS (black), 20 μM TATMYG (blue), and 20 μM TATMYB (red). Error bars represent standard deviations of three biological replicates, with no significant differences noted between treatment conditions.

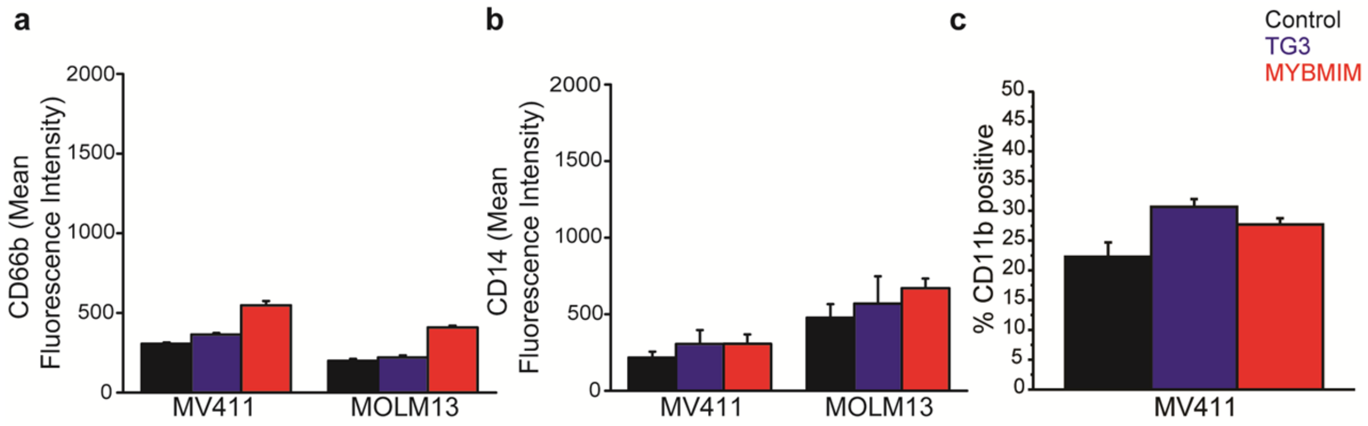

Supplementary Figure 6. Flow cytometry analysis of differentiation of AML cells upon control PBS (black), TG3 (blue), or MYBMIM (red) treatment at 20 μM for 24 hours using CD66b **(a)**, CD14 **(b)**, and CD11b **(c)**, as indicated.

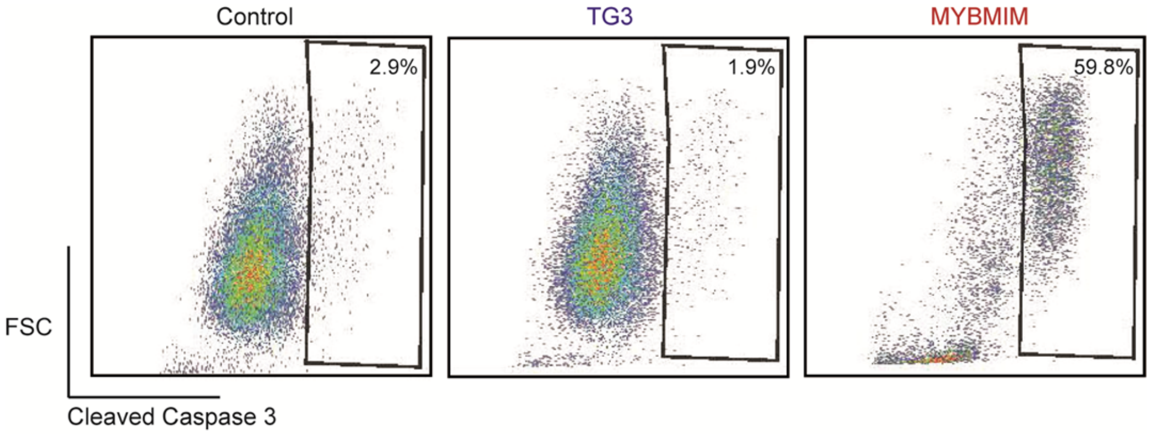

Supplementary Figure 7. Flow cytometry analysis of apoptosis of MV-411 cells upon peptide treatment at 20 μM for 24 hours. Numbers denote percentage of cells that are positive for Cleaved Caspase 3.

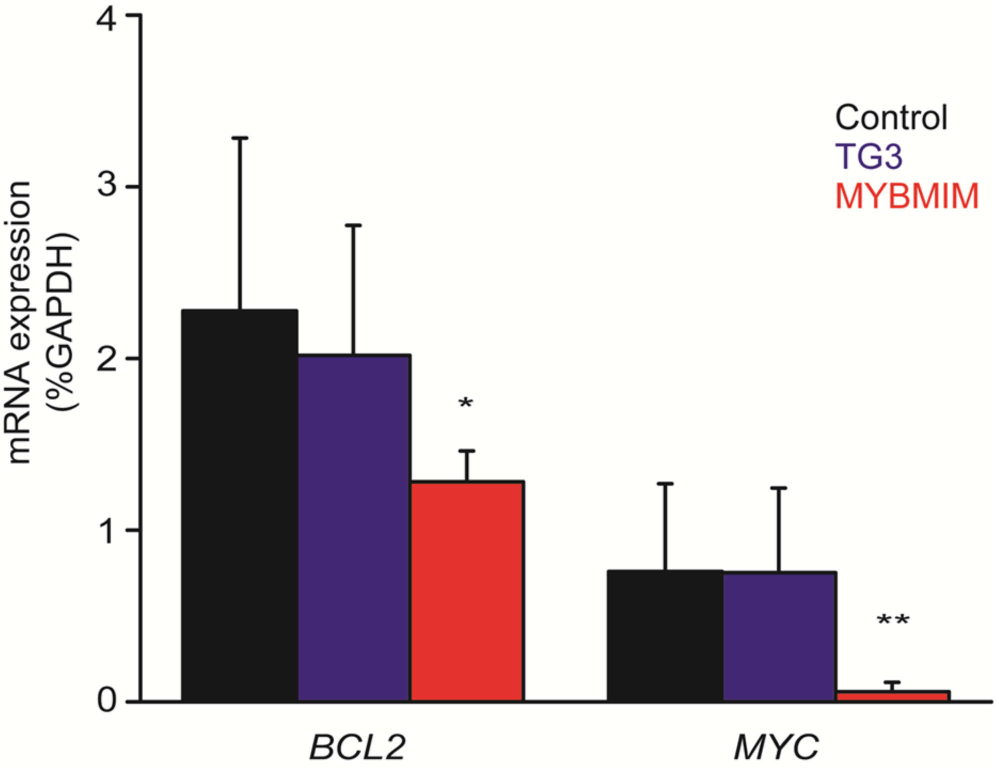

Supplementary Figure 8. Analysis of *BCL2* and *MYC* mRNA expression as measured by qRT-PCR in MOLM-13 cells, upon treatment with control PBS (black), 20 μM TG3 (blue), and 20 μM MYBMIM (red) for 6 hours. Error bars represent standard deviations of three biological replicates. *, $p = 0.0024$; **, $p = 0.077$ when compared to untreated control.

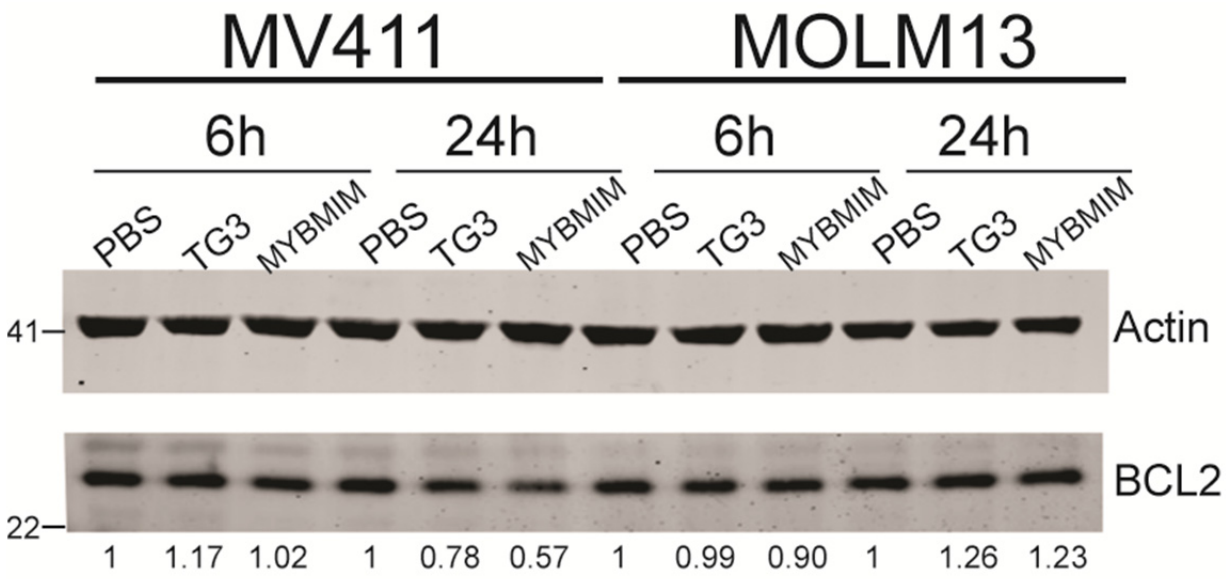

Supplementary Figure 9. BCL2 expression as measured by Western blot in MV411 and MOLM-13 cells, upon treatment with control PBS, 20 μM TG3, and 20 μM MYBMIM for 6 hours and 24 hours. BCL2 band intensities were normalized to their respective actin bands using ImageJ. BCL2 protein expression is quantified as a fraction of its respective PBS control (numbers below blot).

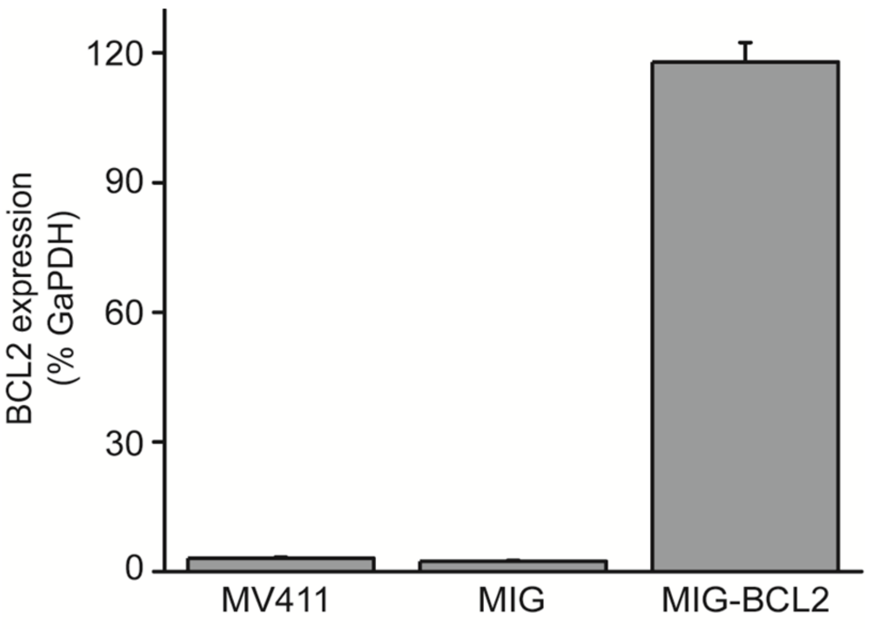

Supplementary Figure 10. Expression of BCL2 in cells retrovirally transduced with MSCV-IRES-GFP vector encoding BCL2. Expression is measured using RT-qPCR and normalized to GAPDH. Error bars represent standard deviations of 3 biological replicates.

Supplementary Table 1: Polypeptide sequences of peptide constructs.

Name	Sequence	Amino acid
FITC-TAT	FITC-AHA-YGRKKRRQRRR-[NH2]	L
TATMYB	[Ac]-YGRKKRRQRRR-GGKRIKELELLLMSTENELK-[NH2]	L
TATMYG	[Ac]-YGRKKRRQRRR-GGKRIKELELLLMSTENGLK-[NH2]	L
MYBMIM	[Ac]-KLENETSMLLLELEKIRKGG-RRRQRRKKRGY-[NH2]	D
TG1	[Ac]-KLENETSMGLLELEKIRKGG-RRRQRRKKRGY-[NH2]	D
TG2	[Ac]-KLGNETSMGLLELEKIRKGG-RRRQRRKKRGY-[NH2]	D
TG3	[Ac]-KLGNETSMGLLELEKIGKGG-RRRQRRKKRGY-[NH2]	D
BIOMYB	[Biotin]-YGRKKRRQRRR-GGKRIKELELLLMSTENELK-[NH2]	L
BIO-MYBMIM	[Ac]-KLENETSMLLLELEKIRKGG-RRRQRRKKRGY[K-Biotin]-[NH2]	D

FITC, fluorescein isothiocyanate; AHA, 6-aminohexanoic acid; Ac, acetyl; Biotin, α-amino biotin; K-Biotin, lysyl ε-amino biotin.

Supplementary Table 2: Significantly downregulated and upregulated gene sets signatures.

Downregulated

Gene set	NES
HALLMARK_MYC_TARGETS_V2	-2.54
SCHUHMACHER_MYC_TARGETS_UP	-2.23
HALLMARK_E2F_TARGETS	-1.99
HESS_TARGETS_OF_HOXA9_AND_MEIS1_UP	-1.86
MULLIGHAN_MLL_SIGNATURE_1_UP	-1.82

Upregulated

Gene set	NES
HALLMARK_TNFA_SIGNALING_VIA_NFKB	2.93
HALLMARK_APOPTOSIS	2.42
HALLMARK_P53_PATHWAY	2.39
HALLMARK_INFLAMMATORY_RESPONSE	2.24
DANG_REGULATED_BY_MYC_DN	2.14

Supplementary Table 3: Genetic characteristics of primary AML used in patient-derived xenograft transplant.

Patient ID	Cytogenetics	Mutations
Patient 11	*MLL-ENL*	*CREBBP*_A467T<br>*KRAS*_G12C<br>*WT1*_R380fs*15<br>*FBXO11*_P49_Q50in

Supplementary Table 4: Oligonucleotide sequences.

Primer	Sequence, 5'-3'
GAPDH, forward	AATCCCATCACCATCTTCCA
GAPDH, reverse	TGGACTCCACGACGTACTCA
BCL2, forward	CTGCACCTGACGCCCTTCACC
BCL2, reverse	CACATGACCCCACCGAACTCAAAGA
MYC, forward	TTCCCCTACCCTCTCAACGACAG
MYC, reverse	CCTCATCTTCTTGTTCCTCCTCAG
BCL2 enhancer, forward	GAGCACAGCGCCAACAGAACTACT
BCL2 enhancer, reverse	TTAAACTGACACGGCTGACTCTCC
NANOG, forward	TCACAAGGGTGGGTCAGTAG
NANOG reverse	CCAGCAGAACGTTAAAATCC

Supplementary Table 5: Body weights for MYBMIM treatment (indicated as a percentage of initial value), corresponding to experiment described in Figure 4I.

**MYBMIM mouse Number**	Treatment Day #1	Day #2	Day #3	Day #4	Day #5	Day #6	Day #7	Day #8	Day #9	Day #10	Day #11	Day #12	Day #13	Day #14
1	1.0	1.0	1.0	1.0	1.0	1.0	1.0	1.1	1.2	1.2	1.2	1.2	1.2	1.2
2	1.2	1.2	1.2	1.2	1.2	1.2	1.2	1.2	1.2	1.2	1.2	1.3	1.3	1.3
3	1.1	1.1	1.1	1.1	1.1	1.1	1.1	1.1	1.1	1.2	1.2	1.3	1.3	1.3
4	1.2	1.2	1.3	1.3	1.3	1.2	1.2	1.2	1.2	1.2	1.2	1.2	1.2	1.2
5	1.3	1.3	1.3	1.3	1.3	1.3	1.3	1.3	1.3	1.3	1.4	1.4	1.4	1.4
6	1.3	1.3	1.3	1.3	1.3	1.3	1.3	1.3	1.4	1.4	1.5	1.5	1.5	1.5
7	1.3	1.3	1.3	1.3	1.3	1.3	1.3	1.3	1.3	1.3	1.3	1.3	1.3	1.3
8	1.2	1.2	1.2	1.2	1.2	1.2	1.2	1.2	1.3	1.3	1.3	1.3	1.3	1.4
9	1.1	1.1	1.1	1.1	1.1	1.1	1.1	1.1	1.2	1.4	1.4	1.4	1.4	1.5
10	1.0	1.0	1.0	1.0	1.0	1.0	1.0	1.1	1.1	1.2	1.2	1.2	1.2	1.2
11	1.0	1.0	1.0	1.0	1.0	1.0	1.1	1.1	1.1	1.2	1.2	1.2	1.2	1.3
12	1.2	1.1	1.1	1.2	1.1	1.2	1.1	1.2	1.1	1.2	1.2	1.2	1.2	1.2
13	1.1	1.1	1.1	1.1	1.1	1.1	1.2	1.2	1.2	1.2	1.3	1.3	1.3	1.3
14	1.1	1.1	1.1	1.1	1.2	1.1	1.2	1.2	1.3	1.3	1.3	1.3	1.3	1.4
15	1.1	1.1	1.1	1.1	1.1	1.1	1.2	1.2	1.3	1.4	1.4	1.5	1.5	1.5

**Control mouse Number**	Treatment Day #1	Day #2	Day #3	Day #4	Day #5	Day #6	Day #7	Day #8	Day #9	Day #10	Day #11	Day #12	Day #13	Day #14
1	1.0	1.0	1.0	1.0	1.0	1.0	1.0	1.0	1.0	1.1	1.1	1.1	1.1	1.1
2	1.0	1.0	1.0	1.0	1.0	1.0	1.0	1.0	1.0	1.0	1.0	1.1	1.1	1.1
3	0.9	0.9	0.9	0.9	0.9	0.9	1.0	1.0	1.0	1.1	1.1	1.1	1.1	1.1
4	0.9	0.9	0.9	0.9	0.9	0.9	0.9	0.9	0.9	0.9	0.9	0.9	0.9	0.9
5	0.9	0.9	0.9	0.9	0.9	0.9	0.9	0.9	0.9	0.9	1.0	1.0	1.0	1.0
6	1.1	1.0	1.1	1.0	1.1	1.1	1.1	1.1	1.1	1.1	1.1	1.1	1.1	1.1
7	0.9	0.9	1.0	1.0	1.0	1.0	1.0	1.0	1.0	1.0	1.0	1.0	1.0	1.0
8	0.9	0.9	0.9	0.9	0.9	0.9	0.9	0.9	0.9	1.0	1.0	1.0	1.0	1.0
9	1.0	1.0	1.0	1.0	1.0	1.0	1.0	1.0	1.1	1.1	1.1	1.1	1.1	1.0
10	0.9	0.9	0.9	0.9	0.9	0.9	0.9	0.9	0.9	0.9	0.9	0.9	1.0	1.0
11	1.0	0.9	1.0	1.0	1.0	1.0	1.0	1.0	1.0	1.0	1.0	1.0	1.0	1.0
12	0.9	0.9	0.9	0.9	0.9	0.9	1.0	1.0	1.0	1.0	1.0	0.9	0.9	0.9
13	0.9	0.9	0.9	0.9	0.9	0.9	0.9	0.9	0.9	0.9	0.9	1.0	1.0	1.0
14	0.9	0.9	0.9	0.9	0.9	0.9	0.9	0.9	0.9	0.9	1.0	1.0	1.0	1.0
15	0.9	0.9	0.9	0.9	0.9	0.9	0.9	0.9	0.9	0.9	0.9	1.0	1.0	1.0
