# Supplementary material for: Peptidomimetic blockade of MYB in acute myeloid leukemia
Source: Nat Commun. 2018 Jan 9;9:110. doi: 10.1038/s41467-017-02618-6 (PMC5760651; doi:10.1038/s41467-017-02618-6)
Supplement: Supplementary file 3 — Description of Additional Supplementary Files [file 41467_2017_2618_MOESM3_ESM.pdf]

Description of Additional Supplementary Files

File Name: Supplementary Data 1
Description: Analysis of gene expression of MOLM13 cells.

File Name: Supplementary Data 2
Description: Analysis of MYB occupancy of MV411 cells.

File Name: Supplementary Data 3
Description: Analysis of H3K27Ac occupancy of MV411 cells.
